# A single high-dose irradiation changes accumulation of methotrexate and gene expression levels of SLC and ABC transporters in cancer cells

**DOI:** 10.3389/fphar.2022.1069321

**Published:** 2023-01-11

**Authors:** Kakeru Sato, Tatsuya Seki, Asuka Mizutani, Yuka Muranaka, Shiho Hirota, Kodai Nishi, Kana Yamazaki, Ryuichi Nishii, Takeo Nakanishi, Ikumi Tamai, Keiichi Kawai, Masato Kobayashi

**Affiliations:** ^1^ Division of Health Sciences, Graduate School of Medical Sciences, Kanazawa University, Kanazawa, Japan; ^2^ Faculty of Health Sciences, Institute of Medical, Pharmaceutical and Health Sciences, Kanazawa University, Kanazawa, Japan; ^3^ Department of Radioisotope Medicine, Atomic Bomb Disease Institute, Nagasaki University, Nagasaki, Japan; ^4^ Department of Molecular Imaging and Theranostics, Institute for Quantum Medical Science, Quantum Life and Medical Science Directorate, National Institutes for Quantum Science and Technology, Chiba, Japan; ^5^ Faculty of Pharmacy, Takasaki University of Health and Welfare, Takasaki, Gunma, Japan; ^6^ Faculty of Pharmaceutical Sciences, Institute of Medical, Pharmaceutical and Health Sciences, Kanazawa University, Kanazawa, Japan; ^7^ Biomedical Imaging Research Center, University of Fukui, Fukui, Japan

**Keywords:** chemoradiotherapy, methotrexate, SLC transporter, ABC transporters, stereotactic body radiotherapy, x-ray, irradiation

## Abstract

Chemoradiotherapy is frequently used to treat cancer. Stereotactic body radiotherapy (SBRT) is a single high-dose radiotherapy used to treat a variety of cancers. The anticancer drug methotrexate (MTX) shows affinity for solute carrier (SLC) and ATP-binding cassette (ABC) transporters. This study investigated relationships between accumulation of methotrexate and gene expression levels of solute carrier and ATP-binding cassette transporters in cancer cells after a single and high-dose X-ray irradiation. Cancer cell lines were selected from lung and cervical cancer cell line that are commonly used for stereotactic body radiotherapy and effective with methotrexate. We examined expression levels of organic anion-transporting polypeptide (OATP)1B1, OATP1B3, OATP1B7, and organic anion transporter (OAT)1 as solute carrier transporters and multidrug resistance-associated protein (MRP)1 and MRP2 as ATP-binding cassette transporters, using real-time polymerase chain reaction and accumulation of ^3^H-MTX in cancer cells after 10-Gy irradiation, assuming stereotactic body radiotherapy. Cells were divided into three groups: Control without irradiation; 4 h after irradiation; and 24 h after irradiation. In control, gene expression levels of OAT1 in all cells was below the limit of measurement. After irradiation, gene expression levels of OATP1B1/1B3/1B7 showed changes in each cell line. Gene expression levels of MRP1/2 tended to increase after irradiation. Gene expression levels of OATP1B1/1B3/1B7 were much lower than those of MRP1/2. Accumulation of ^3^H-MTX tended to decrease over time after irradiation. Irradiation of cancer cells thus alters gene expression levels of both solute carrier transporters (OATP1B1/1B3/1B7) and ABC transporters (MRP1/2) and decreases accumulation of ^3^H-MTX in cancer cells over time due to elevated expression of MRP1/2.

## 1 Introduction

Chemoradiotherapy combines chemotherapy with anticancer drugs and radiotherapy to treat cancer. Chemoradiotherapy can be divided into three categories according to the timing of anticancer drug administration: Neoadjuvant; concurrent; and adjuvant (Baldini et al., 2018). The accumulation of anticancer drugs in cancer cells usually depends on gene expression levels of solute carrier (SLC) and ATP-binding cassette (ABC) transporters (Nakanishi, 2007; Carmichael and Day, 2022). SLC transporters mainly contribute to the uptake of anticancer drugs, while ABC transporters are involved in their excretion (Nakanishi, 2007; Carmichael and Day, 2022).

In radiotherapy, stereotactic radiotherapy involves the delivery of higher doses (7–18 Gy or more) than the usual single-beam dose (1.8–2 Gy) and is used to treat various cancers (Marcrom et al., 2017; Jardel et al., 2020; Sarudis et al., 2021; Ugurluer et al., 2021). Stereotactic radiotherapy was originally used to treat brain cancers, with stereotactic body radiotherapy (SBRT) representing the application of this technology to the trunk of the body, such as for lung and liver cancers (Song et al., 2004; Donovan and Swaminath, 2018; Tandberg et al., 2018; Sarudis et al., 2021; Ugurluer et al., 2021). SBRT has also been shown to be effective against cervical cancer, which is still frequently treated using intracavitary small-source radiotherapy (Ito et al., 2019; Facondo et al., 2021)

Methotrexate (MTX) is a folate antagonist used as an anticancer drug (Visentin et al., 2012). This agent stops cancer growth by preventing the uptake of folic acid, which is necessary in DNA synthesis (Yu et al., 2020). MTX is effective against lung and cervical cancers, where SBRT also appears useful (Conroy et al., 1976; Smyth and Ford, 1981). MTX has shown affinity for the SLC transporters organic anion-transporting polypeptide (OATP) and organic anion transporter (OAT), and the ABC transporters multidrug resistance-associated protein (MRP), multidrug resistance protein (MDR), and breast cancer resistance protein (BCRP) (Hagenbuch and Meier, 2004; Nakanishi, 2007; Murakami and Mori, 2012; Gao et al., 2021). While irradiation increases the expressions of MRP1 and MRP2, contributing to the efflux of MTX (Henness et al., 2002; Bartkowiak et al., 2009), the effects of irradiation on SLC transporters have not been examined. Further, correlations between the kinetics of anticancer drugs including MTX and SLC and ABC transporters after irradiation have yet to be clarified. The purpose of this study was thus to investigate the relationships between accumulation of MTX and expression levels of the genes for SLC and ABC transporters in cancer cells after irradiation. Temporal changes in MTX accumulation in cancer cells after a single and high-dose irradiations assuming SBRT were examined.

## 2 Material and methods

### 2.1 Cancer cell lines

The human-derived lung adenocarcinoma cancer cell lines NCI-H441 (American Type Culture Collection, Manassas, VA, United States) and PC-14 (RIKEN Cell Bank, Tsukuba, Japan) were used. The HeLa human-derived cervical cancer line (RIKEN Cell Bank) was also used. H441, PC-14 and HeLa cell lines were cultivated using RPMI-1640 (FUJIFILM Wako Chemical, Osaka, Japan), Dulbecco’s Modified Eagle’s Medium (FUJIFILM Wako Chemicals) and Eagle’s minimum essential medium (FUJIFILM Wako Chemicals) mixed with 10% fetal bovine serum and 1% sodium pyruvate at 37°C under conditions of 5% CO_2._

### 2.2 Irradiation of cell lines

After achieving 70–80% confluence in a 10-cm diameter plate, each cell line was irradiated with a single 10-Gy X-ray (dose rate, 1.0 Gy/min) using X-ray irradiation equipment (MBR1520R-3; Hitachi, Tokyo, Japan). Cells were divided into three groups: Control without irradiation; 4 h after irradiation; and 24 h after irradiation.

### 2.3 RNA extraction and quality assessment

An RNeasy Plus Mini Kit (QIAGEN, Hilden, Germany) was used to extract RNA from the cancer cells used in this study. The quality of the extracted RNA was evaluated using the RNA integrity number (RIN) as an indicator of quality. The RIN is expressed as a number from 1 to 10, with a higher number reflecting higher quality of RNA. An Affinity Script QPCR cDNA Synthesis kit (Agilent Technologies, Tokyo, Japan) was used for synthesizing cDNA.

### 2.4 Conducting real-time polymerase chain reaction (PCR)

Real-time PCR was performed using an AriaMx 5P system (Agilent Technologies). *OATP1B1/1B3/1B7* as the combination of *OATP1B1*, *OATP1B3* and *OATP1B7* for SLC transporters (because the primer sequences of these transporters are quite similar) and *MRP1* and *MRP2* for ABC transporters were selected as the targets of PCR. The gene *ACBT* for β-actin was used as the internal control gene, as a housekeeping gene that is constantly expressed in all cells. Also, *ACBT* was used to correct for differences in the amounts of initial RNA and cDNA due to differences in sample organization and purification methods. Primer design was outsourced to Eurofins Genomics (Tokyo, Japan). Preparation of cloned plasmids used for the creation of standard curves was outsourced to GenScript (Tokyo, Japan). Primer sequences and concentrations of the genes used are shown in Table 1. A 20-µL volume of PCR reaction solution contained 10 µL of Brilliant III Ultra-Fast SYBR Green QPCR Master Mix (Agilent Technologies), .4 µL of primer, 1 µL of template (10–50 ng of cDNA or cloned plasmid) and 8.6 µL of nuclease-free water. The thermal profile of reaction conditions was: 95°C hot start for 3 min, then 45 cycles of amplification at 95°C for 5 s and 62°C for 15 s, ending with 95°C for 1 min, 55°C for 30 s and 95°C for 30 s.

**TABLE 1:** Primer sequences and concentrations of the used genes.

Transporters/Housekeeping gene		Gene symbol	Primer sequence		Concentration (nM)
			Forward	Reverse	
OATP1B1/1B3/1B7	OATP1B1	*SLCO1B1*	GCA​CTG​GGT​TTC​CAC​TCA​AT	CAG​TTG​TTG​GTG​GAC​CAC​TTT	200
	OATP1B3	*SLCO1B3*	GCA​ATG​GGT​TTC​CAG​TCA​AT	AGC​TGT​TGG​TGG​ACC​ACT​TC	
	OATP1B7	*SLCO1B7*	GCA​ATC​GGC​TTC​CAT​TCA​AT	AGC​TGT​TGG​TGG​ACC​ACT​TC	
OAT1		*SLC22A6*	GCG​CCT​TTT​TTT​GCC​TTC​T	TTC​CCG​CTT​CCC​ATT​GAT​C	300
MRP1		*ABCC1*	GAC​CAT​GAA​TGT​GCA​GAA​GG	GCC​TCA​TCC​AAC​ACA​AGG​AT	100
MRP2		*ABCC2*	CTG​CGG​CTC​TCA​TTC​AGT​CT	GCC​AAG​TTG​GAT​AGG​GTC​AA	
ACTB		*ACTB*	CCAACCGCGAGAAGATGA	CCA​GAG​GCG​TAC​AGG​GAT​AG	

### 2.5 Accumulation of ^3^H-MTX in cancer cells

Each cell was seeded at 1.0 ×10^5^ cells/well in 12-well plastic plates. At about 24 h after seeding, cells were irradiated and divided into three groups: 4 h after irradiation; 24 h after irradiation; and control without irradiation. Each group was pre-incubated for 5 min in phosphate-buffered saline (PBS), then incubated with ^3^H-MTX (10 kBq/well) for 5, 10, 30, or 60 min. After incubation, cells were washed twice with 600 µL of PBS and lysed by 500 µL of .1 M NaOH. Three hundred microliters of cell lysate were mixed with 5 mL of ULTIMA GOLD (Perkin Elmer, Waltham, MA, United States) and the radioactivity of the mixture was measured using a liquid scintillation counter (LSC-5100; Hitachi Aloka Medical, Tokyo, Japan). The results are shown as the percent injected dose/number of living cells measured by an automatic cell counter (LUNA FX7™; Logo Biosystems, Gyeonggi-do, South Korea).

## 3 Results

All cell lines showed RIN >9, indicating high-quality RNA. Measured expression levels of drug transporter genes in each cell line in the three groups by conducting Real-time PCR are shown in Table 2. For SLC transporters, the total gene expression level of OATP1B1/1B3/1B7 was higher in H441 cells than in PC-14 or HeLa cells, but OAT1 in all cells was below the limit of measurement. After cell irradiation, total gene expression levels of OATP1B1/1B3/1B7 decreased in H441 in comparison to before irradiation (control), but increased slightly in PC-14 and HeLa cells.

**TABLE 2:** Gene expression levels of measured drug transporter in each cell by conducting Real-time PCR.

		Gene expression level (×10^5^)		
Transporter	Condition	H441	PC-14	HeLa
OATP1B1/1B3/1B7	Control	13.0	0.01	0.04
	4 h after irradiation	10.5	0.05	0.06
	24 h after irradiation	10.0	0.04	0.09
MRP1	Control	64.5	56.1	34.5
	4 h after irradiation	50.3	78.9	51.0
	24 h after irradiation	79.3	90.3	70.8
MRP2	Control	0.30	1.80	56.7
	4 h after irradiation	0.40	2.30	60.2
	24 h after irradiation	0.80	2.70	98.0
MRP1/2	Control	64.8	57.9	91.2
	4 h after irradiation	50.7	81.2	111.2
	24 h after irradiation	80.1	93.0	168.8

For ABC transporters, expression levels of MRP1/2, as the combination of MRP1 and MRP2, were higher than levels of OATP1B1/1B3/1B7 and OAT1 in control samples of all cancer cells. In control samples, MRP1/2 showed higher expression in HeLa than in H441 or PC-14. In addition, H441 and PC-14 showed higher gene expression levels of MRP1 than MRP2, while HeLa displayed higher gene expression levels of MRP2 than MRP1. After irradiation, gene expression levels of MRP1/2 tended to increase over time in all cancer cell lines.

**FIGURE 1:**
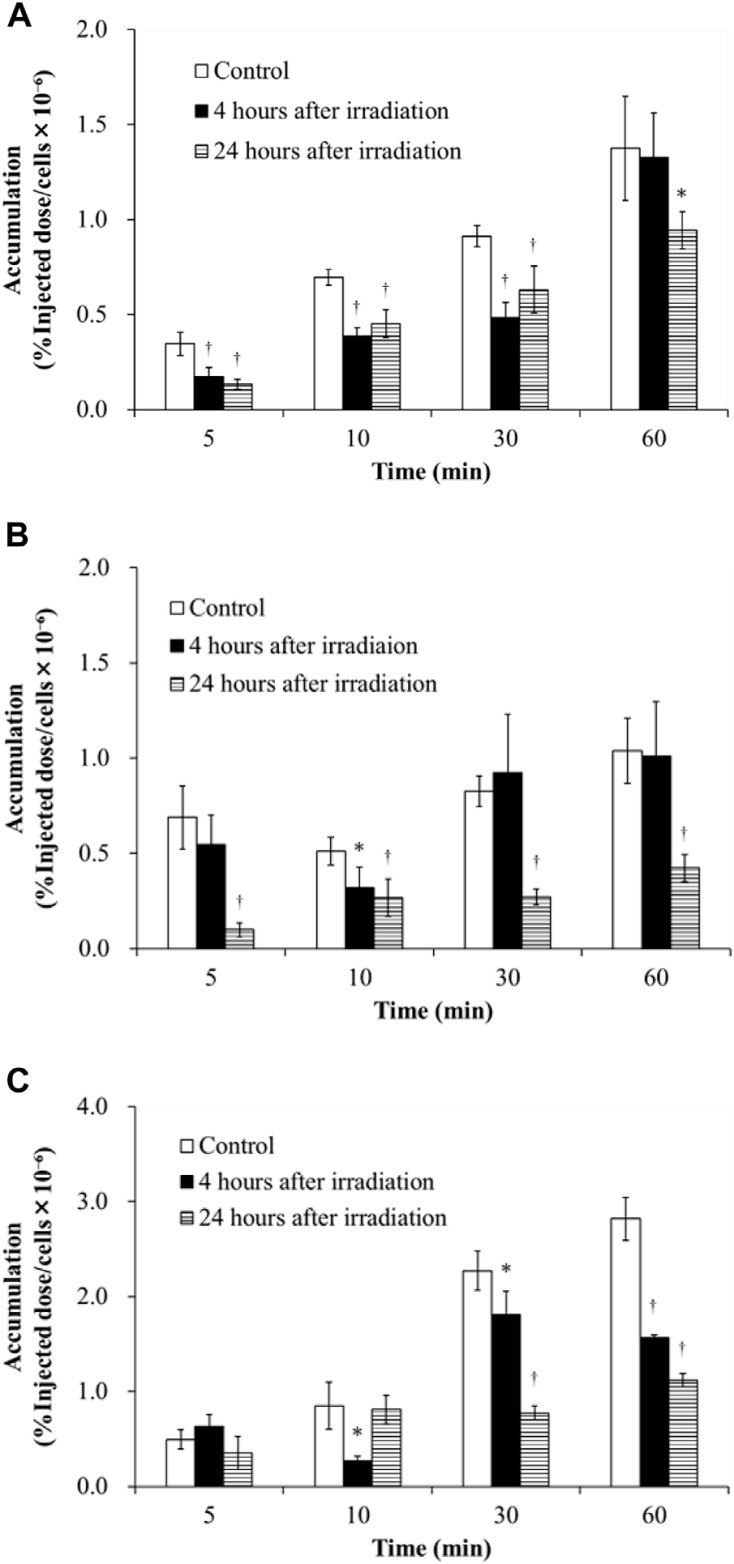
Accumulation of ^3^H-MTX in H441 **(A)**, PC-14 **(B)** and HeLa **(C)** cells in the three groups including control, 4 h after irradiation and 24 h after irradiation at 5, 10, 30, and 60 min after adding ^3^H-MTX. Accumulation of ^3^H-MTX tended to decrease after irradiation in all three cancer cells. ^†^ *p* < .01 and *p < .05 vs. control.

Figure 1 shows the accumulation of ^3^H-MTX in H441, PC-14 and HeLa cells in the control, 4 h after irradiation and 24 h after irradiation groups at 5, 10, 30, and 60 min after ^3^H-MTX injection. Accumulation of ^3^H-MTX was decreased at 4 and 24 h after irradiation in H441 cells and at 24 h after irradiation in PC-14 cells. In HeLa cells, accumulation of ^3^H-MTX was significantly decreased compared to control from 10 min after ^3^H-MTX injection in the 4 h after irradiation group and from 30 min after ^3^H-MTX injection in the 24 h after irradiation group.

## 4 Discussion

Consideration of the effects of irradiation on the kinetics of anticancer drugs in chemoradiotherapy is important. This study examined how the accumulation of ^3^H-MTX in cancer cells was impacted by the effects of gene expression levels for SLC and ABC transporters after X-ray irradiation. Since the degree to which gene expression levels of SLC and ABC transporters are changed under the influence of irradiation was unknown, we examined these gene expressions after irradiation by conducting Real-time PCR (Table 2). Total gene expression levels of OATP1B1/1B3/1B7 decreased after irradiation in H441 cells compared to control, but increased slightly in PC-14 and HeLa cells. For ABC transporters, total gene expression levels of MRP1/2 were higher than OATP1B1/1B3/1B7 and OAT1 in all cancer cell lines under the control conditions. Accumulation of ^3^H-MTX tended to decrease over time after irradiation in all cancer cell lines (Figure 1), and accumulation was significantly decreased at 24 h.

The correlation between accumulation of ^3^H-MTX and gene expression levels of drug transporters is discussed for each cell line. In H441 cells (Table 2), gene expression levels of OATP1B1/1B3/1B7 decreased over time, and MRP1/2 was slightly decreased at 4 h after irradiation in comparison with control and increased further at 24 h after irradiation. Accumulation of ^3^H-MTX in H441 cells was significantly decreased compared to control at 5, 10, and 30 min after adding ^3^H-MTX (Figure 1A). Although we selected MRP1/2 as representative ABC transporters for MTX, changes in MDR and BCRP gene expressions might also influence the accumulation of ^3^H-MTX in H441 (Ji et al., 2013). Henness et al. (2002) reported that expressions of MRP1 and MRP2 were increased after fractionated irradiation, but these expression levels might change over time after a single high-dose irradiation. In PC-14 and HeLa cells (Table 2), gene expression levels of MRP1/2 were much greater than those of OATP1B1/1B3/1B7. After irradiation, the difference between OATP1B1/1B3/1B7 and MRP1/2 became greater over time.

In PC-14 (Figure 1B), accumulation of ^3^H-MTX showed little change between control and 4 h after irradiation, but was significantly decreased at all time points after adding ^3^H-MTX in cells at 24 h after irradiation. Since gene expression levels of OATP1B1/1B3/1B7 were slightly greater in PC-14 cells, the effects on gene expression levels of OATP1B1/1B3/1B7 may be greater than the effects on accumulation of ^3^H-MTX at 4 h after irradiation. At 24 h after irradiation, a correlation was noted between decrease in accumulation of ^3^H-MTX and much higher gene expression levels of MRP1/2.

In HeLa cells (Figure 1C), accumulation of ^3^H-MTX was significantly decreased compared to control at 4 h after irradiation from 10 min after adding ^3^H-MTX, and at 24 h after irradiation from 30 min after adding ^3^H-MTX. Although the effects of drug transporters are usually seen at 5 min after adding ^3^H-MTX, no significant differences at this time points were seen between control and groups at 4 and 24 h after irradiation. An equilibrium state appears to exist between functions of OATP1B1/1B3/1B7 and MRP1/2 at around 5 min after adding ^3^H-MTX, but gene expression levels of MRP1/2 were higher than those of OATP1B1/1B3/1B7 (Table 2). With greater expression of MRP1/2 over time, accumulation of ^3^H-MTX decreased significantly compared to control (Figure 1C). From 10 min after adding ^3^H-MTX, accumulation of ^3^H-MTX was higher in HeLa than in H441 and PC-14. Gene expression levels of MRP2 were also higher in HeLa than in H441 or PC-14, and expression of MRP2 was also higher than that of MRP1. These results may suggest that ^3^H-MTX has higher affinity for MRP1 than for MRP2.

This study was performed assuming SBRT, in which a single exposure provides a higher dose than conventional radiotherapy (Marcrom et al., 2017; Jardel et al., 2020; Sarudis et al., 2021; Ugurluer et al., 2021). Since Lei et al. (2021) reported that the survival rate of HeLa was less than 50% after a single 10-Gy irradiation, we selected a single high-dose of 10-Gy irradiation. In our experiments, cancer cells after a single high-dose irradiation have shown a tendency to excrete anticancer drugs as a foreign body. Neoadjuvant chemotherapy, which administers anticancer drugs prior to radiation, may therefore prove effective in the combination of SBRT and chemotherapy. However, these results only reflect temporal changes following a single irradiation. Future experiments will need to consider fractional irradiation at high dose. In addition, *in vivo* experiments will be required to confirm our *in vitro* results for the accumulation of ^3^H-MTX and expression of drug transporters.

For a more detailed examination, next-generation sequencers might be useful in the future because this method is capable of comprehensively quantifying multitude of various genes (Slatko et al., 2018). However, we intentionally selected MTX which has affinity primarily for OATP and MRP transporters as an anti-cancer drug. Therefore, Real-time PCR, which can accurately quantify gene expression levels of specific transporters, would be appropriate in this study. The Real-time PCR was also used in the study of (Sutherland et al., 2020). They examined the relationship between accumulation of anti-cancer drugs in cancer cells and gene expression levels of specific SLC transporters by Real-time PCR.

## 5 Conclusion

X-ray irradiation with a single, high dose to cancer cells alters gene expression levels of both SLC transporters (OATP1B1/1B3/1B7) and ABC transporters (MRP1/2). In particular, changes in MRP1/2 were much greater than those in OATP1B1/1B3/1B7. Irradiation decreased accumulation of ^3^H-MTX in cancer cells over time due to higher expression of MRP1/2.

## Data Availability

The original contributions presented in the study are included in the article/supplementary materials, further inquiries can be directed to the corresponding author.
